# Supplementary material for: Modeling glioblastoma heterogeneity as a dynamic network of cell states
Source: Mol Syst Biol. 2021 Sep 16;17(9):e10105. doi: 10.15252/msb.202010105 (PMC8444284; doi:10.15252/msb.202010105)
Supplement: Supplementary file 5 — Source Data for Figure 3 [file MSB-17-e10105-s001.zip › Figure3A_sourcedata/GSEA_3065/nosigdb_state5.GseaPreranked.1622718435914/FELDMAN_2020_M-EARLYG1.html]

Details for gene set FELDMAN\_2020\_M-EARLYG1[GSEA]

|  || Dataset | state5 |
| Phenotype | NoPhenotypeAvailable |
| Upregulated in class | na\_neg |
| GeneSet | FELDMAN\_2020\_M-EARLYG1 |
| Enrichment Score (ES) | -0.4970891 |
| Normalized Enrichment Score (NES) | -1.7398758 |
| Nominal p-value | 0.0012690355 |
| FDR q-value | 0.0041973265 |
| FWER p-Value | 0.119 |
Table: GSEA Results Summary

  

Fig 1: Enrichment plot: FELDMAN\_2020\_M-EARLYG1      
 Profile of the Running ES Score & Positions of GeneSet Members on the Rank Ordered List

  

| PROBE | GENE SYMBOL | GENE\_TITLE | RANK IN GENE LIST | RANK METRIC SCORE | RUNNING ES | CORE ENRICHMENT || 1 | MALAT1 |  |  | 0 | 1.196 | 0.0294 | No |
| 2 | PTPRZ1 |  |  | 12 | 0.862 | 0.0493 | No |
| 3 | PTN |  |  | 19 | 0.824 | 0.0689 | No |
| 4 | HSP90B1 |  |  | 33 | 0.729 | 0.0853 | No |
| 5 | SCRG1 |  |  | 36 | 0.715 | 0.1026 | No |
| 6 | GPM6B |  |  | 41 | 0.702 | 0.1194 | No |
| 7 | TNC |  |  | 45 | 0.680 | 0.1358 | No |
| 8 | FGFBP3 |  |  | 85 | 0.545 | 0.1448 | No |
| 9 | PRCP |  |  | 103 | 0.523 | 0.1557 | No |
| 10 | TTYH1 |  |  | 141 | 0.489 | 0.1635 | No |
| 11 | CLU |  |  | 162 | 0.474 | 0.1729 | No |
| 12 | GGH |  |  | 184 | 0.453 | 0.1817 | No |
| 13 | CTGF |  |  | 199 | 0.440 | 0.1909 | No |
| 14 | PSAP |  |  | 227 | 0.420 | 0.1982 | No |
| 15 | ARL6IP1 |  |  | 232 | 0.418 | 0.2080 | No |
| 16 | SPARC |  |  | 327 | 0.370 | 0.2065 | No |
| 17 | TM7SF2 |  |  | 352 | 0.360 | 0.2127 | No |
| 18 | IGFBP7 |  |  | 424 | 0.333 | 0.2129 | No |
| 19 | TNFRSF12A |  |  | 615 | 0.282 | 0.1984 | No |
| 20 | ASPM |  |  | 980 | 0.213 | 0.1626 | No |
| 21 | PLP1 |  |  | 1022 | 0.208 | 0.1631 | No |
| 22 | PCSK1N |  |  | 1162 | 0.190 | 0.1521 | No |
| 23 | SERPINE1 |  |  | 1221 | 0.182 | 0.1500 | No |
| 24 | DKK1 |  |  | 1388 | 0.163 | 0.1353 | No |
| 25 | CENPF |  |  | 1531 | 0.149 | 0.1230 | No |
| 26 | PLD3 |  |  | 1562 | 0.145 | 0.1232 | No |
| 27 | CST3 |  |  | 1602 | 0.141 | 0.1222 | No |
| 28 | MOXD1 |  |  | 1639 | 0.139 | 0.1216 | No |
| 29 | CYR61 |  |  | 1850 | 0.123 | 0.1009 | No |
| 30 | HNRNPA2B1 |  |  | 1852 | 0.122 | 0.1038 | No |
| 31 | IGFBP3 |  |  | 2037 | 0.109 | 0.0858 | No |
| 32 | MEST |  |  | 2059 | 0.108 | 0.0860 | No |
| 33 | GLIPR1 |  |  | 2261 | 0.094 | 0.0657 | No |
| 34 | PLAU |  |  | 3407 | 0.031 | -0.0625 | No |
| 35 | CALD1 |  |  | 4714 | -0.022 | -0.2091 | No |
| 36 | CDK1 |  |  | 4766 | -0.025 | -0.2142 | No |
| 37 | TROAP |  |  | 4869 | -0.028 | -0.2250 | No |
| 38 | C16orf45 |  |  | 5278 | -0.043 | -0.2699 | No |
| 39 | CCNB2 |  |  | 5382 | -0.048 | -0.2803 | No |
| 40 | CCNA2 |  |  | 5671 | -0.061 | -0.3113 | No |
| 41 | TOP2A |  |  | 5722 | -0.063 | -0.3154 | No |
| 42 | KPNA2 |  |  | 5811 | -0.066 | -0.3237 | No |
| 43 | NEXN |  |  | 6052 | -0.077 | -0.3488 | No |
| 44 | LIMA1 |  |  | 6339 | -0.092 | -0.3788 | No |
| 45 | CENPA |  |  | 6374 | -0.094 | -0.3803 | No |
| 46 | TAGLN |  |  | 6733 | -0.116 | -0.4178 | No |
| 47 | HSP90AA1 |  |  | 6962 | -0.134 | -0.4402 | No |
| 48 | AURKA |  |  | 6990 | -0.136 | -0.4399 | No |
| 49 | C4orf48 |  |  | 7110 | -0.146 | -0.4497 | No |
| 50 | CENPW |  |  | 7116 | -0.147 | -0.4467 | No |
| 51 | HNRNPH3 |  |  | 7361 | -0.176 | -0.4698 | No |
| 52 | SNRPA1 |  |  | 7376 | -0.178 | -0.4671 | No |
| 53 | RSU1 |  |  | 7513 | -0.192 | -0.4777 | No |
| 54 | CENPN |  |  | 7528 | -0.194 | -0.4745 | No |
| 55 | UBE2T |  |  | 7561 | -0.199 | -0.4732 | No |
| 56 | BEX4 |  |  | 7566 | -0.199 | -0.4687 | No |
| 57 | BIRC5 |  |  | 7596 | -0.204 | -0.4670 | No |
| 58 | PDLIM7 |  |  | 7864 | -0.247 | -0.4910 | Yes |
| 59 | PSME2 |  |  | 7885 | -0.252 | -0.4871 | Yes |
| 60 | HNRNPM |  |  | 7932 | -0.262 | -0.4858 | Yes |
| 61 | CKS2 |  |  | 7972 | -0.270 | -0.4836 | Yes |
| 62 | HMGB2 |  |  | 7973 | -0.271 | -0.4770 | Yes |
| 63 | FAM162A |  |  | 7983 | -0.272 | -0.4713 | Yes |
| 64 | TPM1 |  |  | 8012 | -0.278 | -0.4676 | Yes |
| 65 | MAP3K7CL |  |  | 8063 | -0.288 | -0.4662 | Yes |
| 66 | DTYMK |  |  | 8075 | -0.292 | -0.4602 | Yes |
| 67 | CDKN3 |  |  | 8079 | -0.293 | -0.4534 | Yes |
| 68 | TMEM14B |  |  | 8105 | -0.298 | -0.4489 | Yes |
| 69 | SAT1 |  |  | 8162 | -0.314 | -0.4475 | Yes |
| 70 | HMGN1 |  |  | 8192 | -0.324 | -0.4428 | Yes |
| 71 | ILF2 |  |  | 8220 | -0.331 | -0.4377 | Yes |
| 72 | HNRNPR |  |  | 8231 | -0.334 | -0.4307 | Yes |
| 73 | LSM5 |  |  | 8242 | -0.337 | -0.4235 | Yes |
| 74 | LDHA |  |  | 8263 | -0.343 | -0.4174 | Yes |
| 75 | UBE2C |  |  | 8278 | -0.348 | -0.4104 | Yes |
| 76 | KHDRBS1 |  |  | 8280 | -0.348 | -0.4020 | Yes |
| 77 | HSPA8 |  |  | 8339 | -0.366 | -0.3995 | Yes |
| 78 | TUBB4B |  |  | 8356 | -0.371 | -0.3922 | Yes |
| 79 | HOXB2 |  |  | 8363 | -0.375 | -0.3837 | Yes |
| 80 | HSPD1 |  |  | 8418 | -0.393 | -0.3801 | Yes |
| 81 | PTTG1 |  |  | 8446 | -0.406 | -0.3732 | Yes |
| 82 | CKB |  |  | 8448 | -0.407 | -0.3633 | Yes |
| 83 | LMNA |  |  | 8478 | -0.423 | -0.3562 | Yes |
| 84 | NUCKS1 |  |  | 8502 | -0.435 | -0.3481 | Yes |
| 85 | C12orf75 |  |  | 8546 | -0.456 | -0.3417 | Yes |
| 86 | GLIPR2 |  |  | 8547 | -0.457 | -0.3305 | Yes |
| 87 | HNRNPA1 |  |  | 8570 | -0.469 | -0.3215 | Yes |
| 88 | HMGN2 |  |  | 8608 | -0.496 | -0.3135 | Yes |
| 89 | HMGB1 |  |  | 8653 | -0.527 | -0.3055 | Yes |
| 90 | HMGA1 |  |  | 8675 | -0.543 | -0.2945 | Yes |
| 91 | H2AFV |  |  | 8693 | -0.554 | -0.2828 | Yes |
| 92 | MLLT11 |  |  | 8695 | -0.555 | -0.2693 | Yes |
| 93 | MT2A |  |  | 8740 | -0.598 | -0.2596 | Yes |
| 94 | H2AFZ |  |  | 8774 | -0.630 | -0.2478 | Yes |
| 95 | STMN1 |  |  | 8783 | -0.638 | -0.2331 | Yes |
| 96 | PHLDA1 |  |  | 8805 | -0.655 | -0.2193 | Yes |
| 97 | FABP7 |  |  | 8811 | -0.661 | -0.2037 | Yes |
| 98 | MZT2B |  |  | 8816 | -0.665 | -0.1878 | Yes |
| 99 | BEX1 |  |  | 8833 | -0.689 | -0.1727 | Yes |
| 100 | HIST1H4C |  |  | 8850 | -0.710 | -0.1570 | Yes |
| 101 | LSM4 |  |  | 8853 | -0.712 | -0.1398 | Yes |
| 102 | COX8A |  |  | 8862 | -0.724 | -0.1229 | Yes |
| 103 | PTMA |  |  | 8894 | -0.764 | -0.1077 | Yes |
| 104 | UBE2S |  |  | 8901 | -0.772 | -0.0894 | Yes |
| 105 | S100B |  |  | 8908 | -0.784 | -0.0708 | Yes |
| 106 | OST4 |  |  | 8925 | -0.829 | -0.0522 | Yes |
| 107 | TUBA1B |  |  | 8980 | -1.103 | -0.0312 | Yes |
| 108 | FTL |  |  | 8984 | -1.286 | -0.0000 | Yes |
Table: GSEA details [plain text format]

  

Fig 2: FELDMAN\_2020\_M-EARLYG1: Random ES distribution      
 Gene set null distribution of ES for **FELDMAN\_2020\_M-EARLYG1**
